# Synergistic Effects of Propolis Combined with 2-Phenoxyethanol and Antipyretics on the Growth of *Staphylococcus aureus*

**DOI:** 10.3390/pharmaceutics13020215

**Published:** 2021-02-04

**Authors:** Katarzyna Grecka, Piotr Szweda

**Affiliations:** Department of Pharmaceutical Technology and Biochemistry, Faculty of Chemistry, Gdańsk University of Technology, ul. G. Narutowicza 11/12, 80-233 Gdańsk, Poland

**Keywords:** propolis, synergism, acetaminophen, 2-phenoxyethanol, ibuprofen, chlorhexidine, octenidine dihydrochloride, acetylsalicylic acid, *Staphylococcus aureus*

## Abstract

The present investigation aimed to assess the combinational effect of commonly used antipyretics and antiseptics with ethanolic extracts of propolis (EEPs) on the growth inhibition of *Staphylococcus aureus*. The broth microdilution checkerboard assay revealed synergistic interactions between all investigated antipyretics, namely acetylsalicylic acid, ibuprofen, and acetaminophen, with EEPs samples. The values of the fractional inhibitory concentration (ΣFIC) index for all these combinations were <0.5. While, in the case of considered antiseptics, namely chlorhexidine, octenidine dihydrochloride, and 2-phenoxyethanol, the positive interaction was confirmed only for the last one (values of ΣFIC in the range 0.0625–0.25). Combinations of two other agents with all four samples of EEPs resulted in an important antagonistic effect (values of ΣFIC ≥ 4.5). Propolis is mostly dedicated to the treatment of skin/wound infections; thus, these findings are of particular practical importance. The outcomes of the study also support the hypothesis that the propolis’s antimicrobial effect is due to the combined (synergistic) action of several ingredients rather than the presence of one component of high antibacterial activity. The composition of 13 ingredients of EEPs (at a concentration below the MIC (minimum inhibitory concentration) of the most active agent) exhibited considerably high anti-staphylococcal efficiency with MIC = 128 µg/mL.

## 1. Introduction

Antibiotic resistance has been reported by the World Health Organization (WHO) as one of the most severe threats to public health [1]. The increasing phenomenon of drug resistance makes modern antibiotics ineffective, so they quickly lose their use in medical practice [2]. Many bacteria strains are resistant to more than one antibiotic, and this phenomenon is called multiple drug resistance. One of the most problematic multidrug-resistant bacteria is methicillin-resistant *Staphylococcus aureus* (MRSA). *S. aureus* is considered to be the most common pathogen and mortality factor in both hospital and non-hospital environments worldwide. It causes a broad spectrum of diseases, ranging from relatively benign skin and soft tissue infections to severe postoperative wound infections and life-threatening conditions, such as sepsis, endocarditis, osteomyelitis, and pneumonia [3,4]. Additionally, *S. aureus* strains produce staphylococcal toxins, causing severe illnesses, such as burned skin syndrome, toxic shock syndrome [5,6], and food poisoning [7].

Due to serious infectious diseases and the continuous development of drug resistance, there is an urgent need to discover new antibacterial substances and/or improve the existing ones [8]. A promising approach to the fight against drug resistance seems to be the search for and research into new, alternative sources of antibacterial substances. Such compounds may inhibit bacterial growth through different action mechanisms than the currently used antibiotics, ensuring effectiveness in fighting infections caused by drug-resistant microorganisms [9,10].

Over the last few years, there has been a renewed increase in interest in the antimicrobial activity of natural bee products; among them, propolis seems to exhibit the most promising therapeutic potential [11,12]. Propolis is a complex resinous mixture collected by honey bees from some trees’ buds and partly enriched with wax, pollen, bee gland secretion, and various mechanical impurities, such as dust, and pieces of wings, and legs of bees, and other insects. Due to its waxy nature and mechanical properties, propolis is used by honey bees as a building material to strengthen the hive’s structure and defend the colony against infections. Propolis is a highly complex biological mixture. Raw propolis usually consists of 50% plant resins, 30% waxes, 10% essential and aromatic oils, 5% pollen, and 5% other organic substances [13]. Propolis’s chemical composition strongly depends on the geographical region, seasonality, and plant species used to produce propolis [14]. In Central European countries, including Poland, bees collect secretions mainly from poplar (*Populus* spp.) and alder (*Alnus* spp.) buds [15]. It contains over a dozen active substances, including flavonoids (flavonols, flavones, and flavanones), aromatic acids, esters, aldehydes, coumarins, terpenes, sterols, fatty acids, and the following microelements: Mn, Fe, Si, Mg, Zn, and Se [13,16,17]. Propolis’s antimicrobial property has been widely investigated, and several authors have demonstrated its antibacterial [17,18,19] and antifungal activity [20,21]. It is believed that propolis’s antibacterial effect results from flavonoids, aromatic acids, and sesquiterpenes [22,23,24].

Numerous literature reports indicate the synergistic effect of propolis and many antibiotics [16,24,25,26,27,28,29,30,31,32,33]. Studies show that propolis reverses resistance to antibiotics whose mechanisms of action consist of inhibiting cell wall synthesis and that it has a synergistic effect with antibiotics that target ribosomes [16]. In our previous studies, we observed the synergistic effect of propolis’s ethanolic extract with (fractional inhibitory index (ΣFIC) ≤ 0.5) aminoglycoside antibiotics (amikacin, kanamycin, and gentamicin), tetracycline, and fusidic acid on the growth inhibition of *S. aureus* [17]. Synergistic interaction between propolis and other antibiotic or non-antibiotic compounds can potentially prevent resistance, increase antibacterial efficacy, and provide broader-spectrum antibacterial activity than antibiotic monotherapy.

Apart from antibiotics, an important group of agents used for the treatment and prophylaxis of bacterial, including staphylococcal, skin infections is disinfectants. Historically, the most common disinfectants were solutions of hydrogen peroxide and tinctures of elemental iodine. Both these agents, however, very effective in the elimination of pathogenic bacteria, exhibit some important drawbacks, including high cytotoxicity (H_2_O_2_ solutions), excessive staining, burning sensation, and potential negative impact on the thyroid (iodine tinctures) [34]. Currently, protection against skin infections is rather performed with disinfectants that contain ingredients that have no side effects. Among them, chlorhexidine, octenidine dihydrochloride, and 2-phenoxyethanol belong to the most common. Moreover, 2-phenoxyethanol may also be utilized in cosmetics and pharmaceuticals as a preservative. Propolis belongs to the most popular alternative/non-antibiotic agents proposed for the treatment of skin and wound infections. One of the main goals of this investigation was to determine whether the ethanolic extract of propolis affects the antimicrobial effectiveness of modern disinfectants’ ingredients. It must be highlighted that both potential effects of combined therapies carried out with propolis and disinfectants, namely synergism and antagonism, could be important for the final result of the therapy.

Propolis is also widely used for the treatment of colds and sore throats. An important symptom of these bacterial infections is fever, which is treated with antipyretics. Because antipyretics are often co-administered with antimicrobials, it is essential to understand the interactions between these two classes of drugs [35]. It has been found that antipyretics have a synergistic effect on the antibacterial activity of some antibiotics [36]. However, to the author’s best knowledge, no research has evaluated the combined effect of these medicines with alternative antimicrobials, such as propolis.

The present investigation aimed to assess the combinational effect of commonly used antipyretics (acetylsalicylic acid, ibuprofen, and acetaminophen) and antiseptics (chlorhexidine, octenidine dihydrochloride, and 2-phenoxyethanol) with ethanolic extracts of propolis on the growth inhibition of *S. aureus*. The outcomes of the study fulfilled some important gaps in our knowledge about therapeutic properties, advantages, and disadvantages of propolis, which could be important in the clinical scenario. Moreover, we also determined the anti-staphylococcal potential of several flavonoids that have been identified as crucial components of this product from the point of view of its antimicrobial effectiveness.

## 2. Materials and Methods

### 2.1. Chemicals and Reagents

The standard compounds and reagents of resazurin sodium salt, dimethyl sulfoxide, ferulic acid, *p*-coumaric acid, 2-phenoxyethanol, acetylsalicylic acid, ibuprofen, acetaminophen, chlorhexidine, phosphate-buffered saline tablet were purchased from Sigma-Aldrich (Saint Louis, MO, USA). The standards of sakuranetin, pinobanksin, pinocembrin, pinostrombin, galangin, apigenin, chrysin, kaempferol were obtained from ChemFaces Biochemical (Wuhan, China). Ethyl alcohol was from Avantor Performance Materials (Gliwice, Poland). Ultrapure water (18.0 MΩ) was obtained with the Milli-Q Advantage A10 system (Millipore, Billerica, MA, USA). The absorbance of the reaction mixture in resazurin assays was measured using the SPARK^®^ multimode microplate reader (Tecan, Männedorf, Switzerland).

### 2.2. Bacterial Strains and Media

The antimicrobial activity of polyphenols of propolis, antipyretics drugs, and antiseptics was tested against two reference strains of bacteria: *Staphylococcus aureus* ATCC 25923 and *Staphylococcus aureus* ATCC 29213 acquired from the American Tissue Culture Collection (ATCC, Manassas, VA, USA), and five MSSA (methicillin-susceptible *Staphylococcus aureus*) and three MRSA (methicillin-resistant *Staphylococcus aureus*) isolates from patients with different infections. *S. aureus* ATCC 25923 was used for synergistic studies. Bacteria were routinely grown on Luria-Bertani (LB) plates (Saint Louis, MO, USA). The minimum inhibitory concentrations (MICs) were determined using Mueller-Hinton Broth (MHB, Sigma-Aldrich, Saint Louis, MO, USA), and for the determination of minimum bactericidal concentrations (MBCs), the broths used for MIC determination were subcultured onto Baird Parker Agar plates (Biomaxima, Lublin, Poland).

### 2.3. Ethanol Extracts of Propolis and Polyphenols Mixture Preparation

Four raw samples of *Apis mellifera* propolis were obtained from Polish apiaries between autumn and spring of 2015 (EEP2), 2017 (EEP3, EEP4), and 2018 (EEP1). The crude samples were kept in a dry place and stored at room temperature in the dark until processing. Ethanolic extracts of propolis were obtained using the previously described method [17]. Briefly, 5 g of propolis samples were added to 50 mL of 70% ethanol and macerated for 100 h under gentle agitation. After extraction, the ethanol extract solutions were centrifuged at 9000 rpm for 10 min. The collected supernatants were filtrated through 0.22 µm pore size filters, evaporated to dryness by rotary vacuum evaporator, and then solutions of desired concentrations were prepared in 70% ethanol.

Polyphenols were dissolved in dimethyl sulfoxide (DMSO) to final concentrations of 10.24 mg/mL. A total of 13 polyphenols (sakuranetin, pinobanksin, pinocembrin, pinostrombin, galangin, apigenin, chrysin, kaempferol, ferulic acid, isoferulic acid, *p*-coumaric acid, caffeic acid quercetin) were combined together to prepare the mixture of polyphenols. The mixture’s final concentration was prepared at a concentration of 10.24 mg/mL. The concentration of each polyphenol in the mixture was equal. Representatives of polyphenols were chosen based on our previous investigations of the chemical composition of Polish propolis samples [17].

### 2.4. Determination of Minimum Inhibitory Concentration and Minimum Bactericidal Concentration

Minimum inhibitory concentrations (MICs) were determined by the resazurin-based broth microdilution assay [37]. Briefly, several bacterial colonies were picked from an LB plate that had been incubated overnight and suspended in PBS(phosphate-buffered saline). The bacterial cell suspension was adjusted to the optical density (OD) of OD_600_ = 0.1 and further diluted at a ratio of 1:100 *v/v* to ∼10^6^ CFU/mL in MHB. A volume of 100 µL of the suspension was added to 96-well microtiter plates, with each well containing 100 µL of a dilution series of antimicrobials previously prepared in MHB. The plates were incubated overnight at 37 °C. The 30 µL of resazurin sodium salt (0.015% in PBS) was added to each well of a microtiter plate. The plates were further incubated for 90 min at 37 °C in the dark, and MICs were determined through fluorescence measurement at 550 and 590 nm excitation. The lowest concentrations of antimicrobials with no bacterial growth were taken as MIC values. For the determination of minimum bactericidal concentrations (MBCs), a small volume of each dilution used for MIC assay was transferred with a 48-well microtiter plate replicator on Baird-Parker agar plates. The MBC value was where there was no colony growth observed after overnight incubation at 37 °C.

### 2.5. Growth Curve

To obtain the growth kinetics of *S. aureus* ATCC 25923 in the presence of different concentrations of flavonoids, a microtiter plate-based assay was used. Growth curves were performed for four flavonoids: galangin, quercetin, kaempferol, and pinocembrin, which revealed the anti-staphylococcal activity. Two-fold dilutions of flavonoids were prepared in DMSO. A 10 µL of each dilution was added to wells in the microtiter plate. Inoculum of ∼5.0 × 10^5^ CFU/mL was added for a final volume of 200 μL. The final concentrations of each dilution were 1024, 512, 256, 128, 64, 32, 16, 8, 4, and 2 μg/mL. Microbial growth kinetics was recorded for 24 h on the SPARK^®^ multimode microplate reader (Tecan, Männedorf, Switzerland). Every hour, culture turbidity was measured as absorbance at 600 nm, with 10 s agitation before each OD measurement. XY graphs were made using GraphPad Prism^®^ 8.0. 1 (GraphPad Software, Inc., La Jolla, CA, USA).

### 2.6. Checkerboard Dilution Test

Checkerboard assay was performed to determine possible additive/synergistic drug interactions when using combinations of a propolis extract with antiseptics (2-phenoxyethanol, chlorhexidine, and octenidine dihydrochloride) and antipyretics (acetylsalicylic acid, ibuprofen, and acetaminophen) against *S. aureus*. Four samples of propolis (EEP1, EEP2, EEP3, and EEP4) were tested. The test was performed according to the procedure presented in our previous report [17] with some modifications. Briefly, two-fold dilutions of agent A were prepared, and 10 µL of each dilution was distributed along the y-axis in a 96-well plate. Then, 10 µL of previously prepared two-fold dilutions of agent B were distributed along the x-axis. Afterward, each well in the microtiter plate was inoculated with 180 µL of ∼5.0 × 10^5^ CFU/mL inoculum. Final concentrations of both agents ranged from at least MIC to 1/64 MIC. Following the incubation of the plates at 37 °C for 24 h under static conditions, 30 µL of resazurin solution (0.015% in PBS) was added to all wells, and plates were incubated for further 90 min at 37 °C in the dark. After this time, fluorescence was measured at 550 and 590 nm.

### 2.7. Fractional Inhibitory Concentration (FIC) Index Calculation

The data from the checkerboard assay were used for ΣFIC values calculation, and results were analyzed according to the guidelines published by Odds [38]. Fractional inhibitory concentration index (ΣFIC) was calculated for each combination as follows (Equation (1)):

(1) $$\sum \mathrm{FIC}=\frac{\mathrm{MIC}\;\mathrm{of}\;\mathrm{EEP}\;\mathrm{in}\;\mathrm{combination}\;\mathrm{with}\;\mathrm{agent}\;B}{\mathrm{MIC}\;\mathrm{of}\;\mathrm{EEP}\;\mathrm{alone}}+\frac{\mathrm{MIC}\;\mathrm{of}\;\mathrm{agent}\;B\;\mathrm{in}\;\mathrm{combination}\;\mathrm{with}\;\mathrm{EEP}}{\mathrm{MIC}\;\mathrm{of}\;\mathrm{agent}\;B\;\mathrm{alone}}$$

The interaction of two antimicrobial agents was considered synergistic if the ΣFIC was ≤ 0.5, indifferent when the ΣFIC index value was in the range from 0.5 to 4.0, and antagonistic if the ΣFIC was ≥ 4.0.

### 2.8. Synergy Score Calculation

The fluorescence measurement data were also visualized and analyzed using freely available software, Combenefit (version 2.021, Cancer Research UK Cambridge Institute, Cambridge, UK), which simultaneously assesses synergy/antagonism from dose-response data using three classical models, namely the Loewe, the Bliss, and the Highest Single Agent (HAS). In this study, we applied the Loewe additivity theory for two-drug combinations. The analysis was performed as described by Di veroli et al. [39]. Briefly, the data were expressed as a “percentage of control” and saved in .xls files according to a template provided by the software developers. The difference between the Loewe model-based expected additive effect and the actual effect of the drug combination was calculated by the software. This difference value is called a synergy score. The software calculates a synergy score for each combination, where a positive score indicates synergy, a score of 0 is additive, and a negative score indicates antagonism [39]. The calculated values were represented as a synergy heatmap with the color scale from blue (synergism) to red (antagonism).

### 2.9. Data Analysis

All experiments in this study were completed in triplicate, and data were expressed as the means ± SD.

## 3. Results

### 3.1. Antimicrobial Activity of Antipyretics and Antiseptics

The antibacterial activities of antipyretics (ibuprofen, aspirin, and acetaminophen) and antiseptics (2-phenoxyethanol, chlorhexidine, and octenidine dihydrochloride) were tested against two reference and eight clinical staphylococcal strains. The results are shown in Table 1 and Table 2.

All of the antipyretics showed weak antibacterial activity against reference strains and MSSA and MRSA isolates of *S. aureus*. Ibuprofen showed the most potent antibacterial activity, with MICs of 500 µg/mL and MBCs ranging from 1000 to 4000 µg/mL. The MICs of acetylsalicylic acid ranged from 1000 µg/mL to 2000 µg/mL. The MBC of acetylsalicylic acid was found to be 4000 µg/mL. Acetaminophen exhibited an inhibitory effect against five out of ten tested strains with a MIC value of 8000 µg/mL. At the tested concentrations of acetaminophen, no bactericidal effect was observed against all strains.

All tested antiseptics effectively inhibited the growth of all tested strains. MICs of 2-phenoxyethanol ranged from 0.156% to 0.312% (*v/v*), and the values of MBC of 2-phenoxyethanol were in the range from 0.312% to 1.25% (*v/v*). MICs and MBCs of chlorhexidine ranged from 0.1 µg/mL to 0.4 µg/mL. MICs and MBCs of octenidine dihydrochloride ranged between 0.2 and 0.4 µg/mL.

Both reference and clinical *Staphylococcus aureus* strains were relatively equally sensitive to the tested antipyretics and antiseptics. Therefore, the reference *S. aureus* ATCC 25923 strain was selected for the synergism studies.

### 3.2. In Vitro Evaluation of Combinations of Antipyretics and Antiseptics with Propolis Ethanolic Extracts

**Figure 1:**
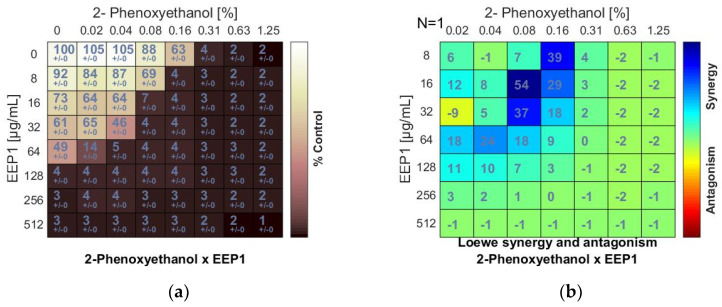
Synergistic effect between ethanolic extract of propolis and 2-phenoxyethanol against *S. aureus* ATCC 25923: (**a**) Checkerboard assay results showing the percentage growth inhibition compared with control; (**b**) Results showing synergy/antagonism calculations of synergy scores determined by the Combenefit software (the difference between the predicted additivity and the observed viability) for the Loewe model. A heat map represents the level of synergy (blue color) at each concentration. A positive score indicates synergy, a score of 0 is additive, and a negative score indicates antagonism (red color).

**Figure 2:**
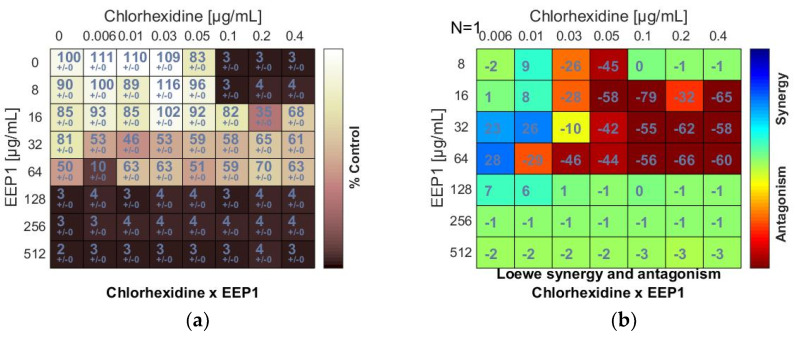

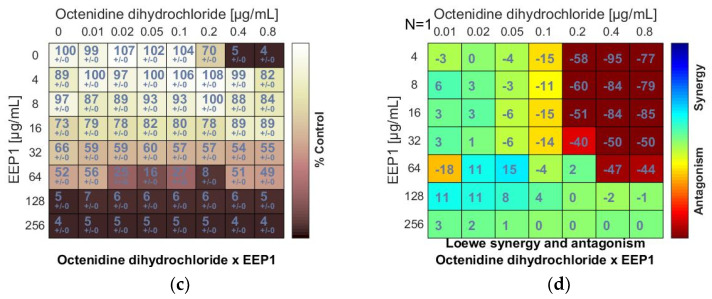
Antagonistic effect between ethanolic extract of propolis and antiseptic (chlorhexidine and octenidine dihydrochloride) against *S. aureus* ATCC 25923: (**a**,**c**) Checkerboard assay results showing the percentage growth inhibition compared with control; (**b**,**d**) Results showing synergy/antagonism calculations of synergy scores determined by Combenefit software (the difference between the predicted additivity and the observed viability) for the Loewe model. A heat map represents the level of synergy (blue color) or antagonism (red color) at each concentration. A positive score indicates synergy, a score of 0 is additive, and a negative score indicates antagonism.

**Figure 3:**
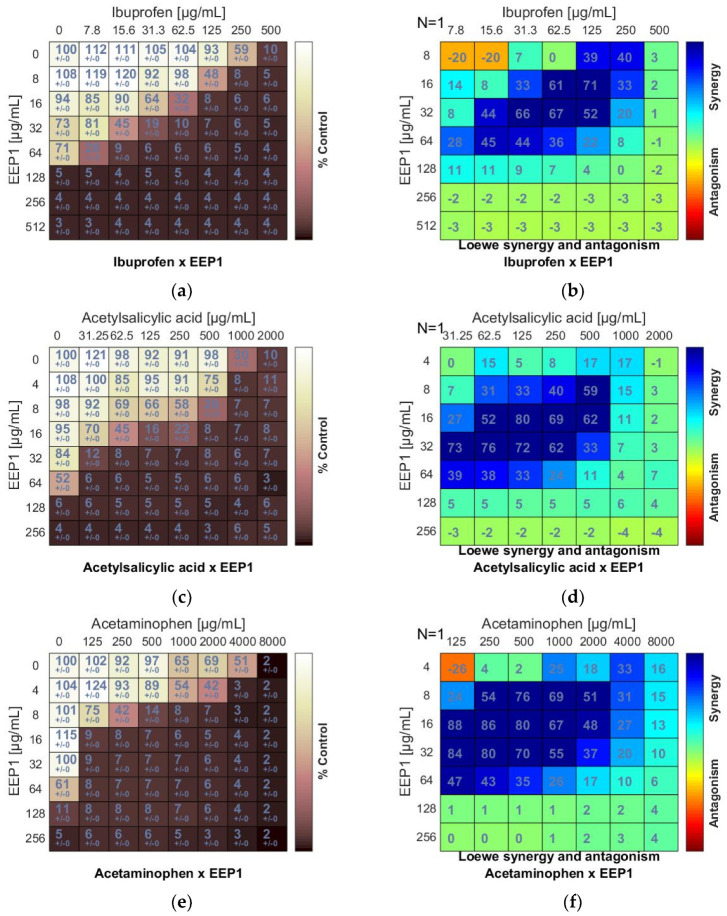
Synergistic effect between ethanolic extract of propolis and antipyretics against *S. aureus* ATCC 25923: (**a**,**c**,**e**) Checkerboard assay results showing the percentage growth inhibition compared with control; (**b**,**d**,**f**) Results showing synergy/antagonism calculations of synergy scores (the difference between the predicted additivity and the observed viability) for the Loewe model. A heat map represents the level of synergy (blue color) at each concentration. A positive score indicates synergy, a score of 0 is additive, and a negative score indicates antagonism (red color).

In the checkerboard assay, antipyretics drugs (ibuprofen, acetylsalicylic acid, and acetaminophen) and antiseptics (2-phenoxyethanol, chlorhexidine, and octenidine dihydrochloride) were combined with four samples of EEPs (EEP1, EEP2, EEP3, EEP4). Combinations were tested on the growth of *S. aureus* ATCC 25923. Concentrations ranged from several dilutions below the MIC to the MIC value or 1–3 dilutions above the MIC. Drug combination effects were assessed on the basis of the values of the calculated fractional inhibitory concentration (ΣFIC) index, as was proposed by Odds [38]. The interaction between two antimicrobial agents was considered synergistic when the ΣFIC was ≤0.5, indifferent when the ΣFIC value was in the range between 0.5 and 4.0, and antagonistic for ΣFIC ≥ 4.0. The synergy level between two drugs was also quantified and visualized by a tool called Combenefit using Loewe additive model. Combenefit provides set of scores that capture information about the synergy distribution (≤1 = synergy, 0 = additive, >1 = antagonism). The ΣFICvalues of the best combinations are listed in Table 3 and Table 4. The checkerboard results showing growth inhibition percentage and heatmaps showing Loewe synergy scores distribution are demonstrated in Figure 1, Figure 2 and Figure 3 (only data for EEP1 are presented).

The minimum inhibitory concentrations (MIC) of EEPs, antiseptics, and antipyretics were previously determined for *S. aureus* ATCC 25923 using broth microdilution assay (Table 1 and Table 2). A synergistic effect was observed when 2-phenoxyethanol (PE) was combined with EEP1, EEP2, EEP3, or EEP4, as demonstrated by the synergy score matrix (Figure 1b) and the mean ΣFIC of 0.375, 0.375, 0.187, and 0.312, respectively (Table 3). The highest synergistic effect (ΣFIC: 0.187) was observed for the PE + EEP3 combination. In this case, the MIC for PE was reduced to 1/8×MIC in the presence of EEP3 at a concentration of 1/16 × MIC.

The chlorhexidine (C) + EEPs combinations showed antagonistic interactions. Loewe synergy and antagonism heat maps (Figure 2b) clearly indicated antagonism (red color). The ΣFIC values calculated for C + EEP1, EEP2, EEP3, and EEP4 combinations ranged from ≥5 to ≥10. The MIC of C was at least two dilutions higher in combination with each EEP than the MIC of C alone. However, C did not increase the MICs of EEPs (Figure 2a,b).

An antagonism was also detected when EEP samples were combined with octenidine dihydrochloride (OD). The negative scores marked in red (Figure 2d) and the ΣFIC values calculated for the combinations of OD and EEP1, EEP2, EEP3, or EEP4 ranged from ≥4.5 to ≥6 indicated antagonism. Similar to chlorhexidine, octenidine dihydrochloride did not affect MICs of EEPs.

As a result of combining antipyretics with EEPs, a synergism was observed for all drug + EEP combinations. The heatmaps represented the distribution of synergism as positive scores marked in blue (Figure 3b,d,f). ΣFIC indices of ibuprofen (IB) and EEP1, EEP2, EEP3, or EEP4 were from 0.187 to 0.375. The most synergistic combination was identified for EEP3 + IB. In this combination, the MIC for IB was reduced to 1/16 × MIC in the presence of EEP3 at a concentration of 1/8 × MIC.

Synergy was evident for acetylsalicylic acid (AS) in combination with all samples of EEPs, as the ΣFIC indices ranged from 0.078 to 0.281. The most potent synergy was observed for the combination of EEP3 + IB. The MIC of IB was reduced to 1/64 × MIC in the presence of EEP3 at a concentration of 1/16 × MIC.

A predominant synergism was also detected when acetaminophen (AM) was combined with EEPs. The ΣFIC indices ranged from 0.094 to 0.312. The best synergy effect was observed for the combination of EEP2 + AM. In this case, the MIC of AM was reduced to 1/32 × MIC in the presence of EEP3 at a concentration of 1/16 × MIC.

### 3.3. Antimicrobial Activity of Flavonoids of Propolis

**Figure 4:**
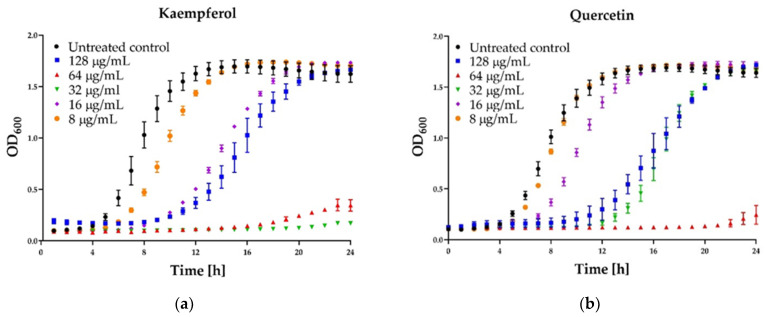

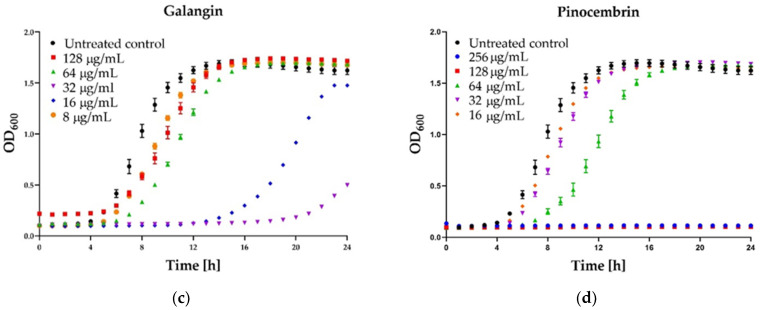
Growth curve assay for selected polyphenols tested against *S. aureus* ATCC 25923 at different concentrations. The growth control contained no antimicrobial compound. (**a**) Kaempferol; (**b**) Quercetin; (**c**) Galangin; (**d**) Pinocembrin. The results are presented as means ± SD (*n* = 3). Data without error bars indicate that the SD is too small to be observed on the graph.

Selected major propolis compounds (flavonols, flavones, flavanones, and phenolic acids) were investigated to evaluate their antibacterial potency against *Staphylococcus aureus* strains as individual compounds and in the mixture. The effects of the 13 polyphenols and polyphenols mixture on the growth of the *S. aureus* ATCC 25923 and *S. aureus* ATCC 29,213 tested are presented in Table 5. The results revealed that six out of thirteen compounds effectively suppressed microbial growth of *S. aureus* reference strains. Flavonols (kaempferol, galangin, and quercetin) were found to be the most active compounds, with MICs ranging between 32 and 64 μg/mL against both *S. aureus* strains. However, in those flavonols, paradoxical growth was observed above their MIC values (Figure 4). Pinocembrin and sakuranetin exhibited slightly lower activity with MIC of 128 and 256 μg/mL against both strains tested, respectively.

The lowest, but still observed, anti-staphylococcal activity was identified for pinobanksin with MIC of 1024 μg/mL. In up to 1024 µg/mL concentration, no activity was observed for phenolic acids (ferulic acid, isoferulic acid, caffeic acid, *p*-coumaric acid) or flavones (apigenin, chrysin). The MIC of the polyphenol mixture was 128 µg/mL for both staphylococcal reference strains. When used individually, polyphenols showed bacteriostatic activity. Almost all MBC values of individual propolis components were above the tested range. Of all the polyphenols tested, the only pinocembrin showed a bactericidal effect with an MBC value of 512 µg/mL in the case of the growth of *S. aureus* 29213. The polyphenols mixture exhibited significantly higher bactericidal efficiency with MBC values of 256 and 1024 µg/mL against reference strains of *S. aureus*. Obtained results were also confirmed for clinical isolates, including five methicillin-susceptible strains (MSSA) and three methicillin-resistant isolates (MRSA). MIC values obtained for clinical isolates were similar to the results observed for reference strains (Table 6), and only the mixture of the agents exhibited bactericidal activity (against seven out of eight strains tested). No differences in the susceptibility were observed between MSSA and MRSA isolates.

Some interesting results were provided by the kinetic assay of the growth of the staphylococcal cells in the medium supplemented with different concentrations (namely 8, 16, 32, 64, 128 µg/mL) of six polyphenols that exhibited activity. In the case of three agents: pinocembrin (Figure 4d), pinobanksin, sakuarentin, the efficiency in growth inhibition was strictly correlated with the concentration of the agents, and an increase in concentration resulted in higher antibacterial activity. A few unexpected results were observed for other substances. In the case of kaempferol (Figure 4a), the highest efficiency in growth inhibition was achieved for concentrations of 32 and 64 µg/mL. In medium supplemented with this agent to the final concentration of 128 µg/mL, the growth was efficiently inhibited only for about 8 h. Subsequently, accelerated growth of bacteria was observed in this medium, and after about 20 h, it was equal to the control (growth in the medium free of antibacterial agents). Quite a similar effect was observed for quercetin (Figure 4b)—concentration of 128 µg/mL exhibited lower activity than 64 µg/mL and comparable to 32 µg/mL. In the case of galangin (Figure 4c), a much better effect of growth inhibition was observed at a concentration of 16 and 32 µg/mL compared to 64 and 128 µg/mL.

## 4. Discussion

The antimicrobial activity of propolis has been extensively investigated in the past decade. Numerous studies have reported that propolis exhibits bacteriostatic activity against various bacterial species. It can be bactericidal in high concentrations, and it is more effective against Gram-positive than Gram-negative bacteria. The mode of action of propolis on bacterial cells is complex and differs from standard antibiotics [40]. According to the latest reports, propolis induces bacterial death through cell lysis, similar to lytic peptides [41]. This antimicrobial effect could be attributed to phenols and flavonoids present in propolis [42]. Our previous study confirmed that propolis samples with high phenolic content exhibit a potent antibacterial effect [17]. Such compounds are bioactive molecules whose biological activity is closely related to the hydroxyl groups or phenolic rings found in their molecular structure [23,43]. Flavonoids, such as galangin, pinocembrin, kaempferol, sakuranetin, and quercetin, and other phenolic compounds, e.g., caffeic acid and its derivatives, are suggested as antimicrobial components of propolis. Mirzoeva et al. [24] found that some isolated propolis components, including cinnamic derivatives and flavonoids, affect the potential and mobility of bacterial membranes. Quercetin has been reported to disrupt cell membranes and inactivate extracellular proteins by forming irreversible complexes [44]. Galangin and caffeic acid derived from propolis are considered to be bacterial enzyme inhibitors [45]. Takaisi-Kikuni and Schilcher [40] revealed that some components of propolis could inhibit bacterial RNA-polymerase and protein synthesis and affect bacterial growth by preventing cell division, causing the formation of pseudomulticellular bacterial forms. From a clinical perspective, identifying and isolating the active compounds of propolis responsible for the inhibitory effects could be useful for developing new antibiotic drugs.

This study evaluated the anti-staphylococcal potential of 13 phenolic compounds, namely sakuranetin, pinobanksin, pinocembrin, pinostrombin, galangin, apigenin, chrysin, kaempferol, quercetin, caffeic acid, *p*-coumaric acid, ferulic acid, and isoferulic acid. The anti-staphylococcal activity was found for three flavanones (pinocembrin, pinobanksin, and sakuranetin) and three flavonols (galangin, kaempferol, and quercetin). However, those flavonols’ antimicrobial effects occurred only at a narrow range of low concentrations, while increased dosage beyond a certain point decreased their effects. This paradoxical “more-kill-less” response is described in the literature as the Eagle effect [46]. Such a phenomenon occurs when high concentrations of antibiotics above the minimum inhibitory concentration (MIC) or minimum bactericidal concentration (MBC) improve the levels of surviving cells. Moreover, MBC and kinetics experiment results showed that the mechanism of antimicrobial action of galangin, kaempferol, and quercetin was rather bacteriostatic than bactericidal. Besides, we evaluated an anti-staphylococcal potential of polyphenols mixture. Its MIC was found to be 128 µg/mL. It was an interesting result of this study because the concentrations of the individual compounds in the mixture were lower than the MIC of the most active compound used separately. This finding supports the speculation that the propolis’s antimicrobial effect is due to the combined (synergistic) action of several ingredients rather than the presence of one component of high antibacterial activity. Moreover, these synergistic interactions resulted also in a bactericidal activity that was not observed for the agents used alone. These observations additionally highlight the advantages of this propolis (but also probably other natural products) compared to its pure ingredients.

Combining antibiotics with activity-enhancing plant-derived compounds is a promising strategy in the treatment of infectious diseases. Combination antibiotic therapy allows for broadening antibacterial spectrum, synergistic effects, and reduced risk for emerging resistance during treatment. Synergism between propolis and antibiotics has been observed. However, little attention has been given previously to the combined effects of propolis and other antimicrobial agents, such as antiseptics. To our knowledge, this is the first time that the effects of propolis and those agents have been investigated. The in vitro combined effect of three antiseptics (i.e., 2-phenoxyethanol, chlorhexidine, octenidine dihydrochloride) was tested.

The checkerboard and Loewe analyses revealed synergism between 2-phenoxyethanol and ethanolic extracts of propolis (EEPs). The antiseptic 2-phenoxyethanol has a broad spectrum of antimicrobial activity and has been used widely in antiseptic products or as a preservative in cosmetics, vaccines, and pharmaceuticals. Phenoxyethanol acts by uncoupling oxidative phosphorylation from respiration and inhibiting malate dehydrogenase by competing for the enzyme’s active site and increasing the cell membrane’s permeability to potassium ion. It is frequently combined with other antibacterial agents to obtain sufficient antimicrobial effect [47]. Taking advantage of EEP’s synergy seems to be a promising approach for keeping the concentration of 2-phenoxyethanol in different products as low as possible. In our opinion, propolis extract used in combination with 2-phenoxyethanol demonstrates the potential to act as a surface-active antibacterial agent or a preservative in products, such as ointments, mouthwash, soaps, and toothpaste.

Another important finding was that antagonistic interactions were observed for chlorhexidine and octenidine dihydrochloride combined with EEPs. All investigated propolis extracts increased MICs of chlorhexidine and octenidine dihydrochloride by at least two dilutions. However, the antiseptics did not seem to interfere with the extracts’ antibacterial effect. Chlorhexidine and octenidine dihydrochloride are antiseptic agents used for skin disinfection. Chlorhexidine and octenidine dihydrochloride are positively charged molecules that attach directly to the negatively charged teichoic and teichuronic acids in the bacterial cell wall, causing bacterial cell membrane disruption and cytoplasmic components’ leakage [48,49]. Flavonoids disrupt microbial membranes and form complexes with extracellular and soluble proteins [23,43]. In the presence of propolis, one may speculate that chlorhexidine and octenidine dihydrochloride cannot be absorbed within the bacterial cell wall structure, which exhibits its antibacterial action. This finding is important from a clinical point of view. Currently, EEP is mostly proposed for the treatment of skin and difficult to heal wound infections. The obtained results clearly indicate that propolis should not be combined with medical products containing these two components: chlorhexidine and octenidine dihydrochloride.

Analgesics, antipyretics, and non-steroidal anti-inflammatory drugs (NSAIDs) are some of the most commonly used medicines. Since they are generally prescribed along with antibiotics to treat infectious diseases, it is crucial to understand the interactions between these two classes of drugs. Recently, there has been a renewed interest in the abilities of treatment of infections with natural products, including propolis. Thus, knowledge about the interactions of this product with antipyretics is of particular importance. Several studies have shown that antipyretics, including NSAIDs, exhibit antiviral, antifungal, and antibacterial properties [35,50,51,52,53]. This study confirmed that ibuprofen, acetylsalicylic acid, and acetaminophen exhibited a weak antimicrobial effect against *S. aureus* strains with MICs ranging from 500 to 8000 µg/mL. These findings are consistent with that of Chan et al. [36], who found that ibuprofen possesses antibacterial activity against *Bacillus sp.* and *S. aureus*, with MICs of 0.625 mg/mL and 2.5 mg/mL, respectively. Chan et al. also reported that acetylsalicylic acid exhibits inhibitory activity against a broad spectrum of Gram-negative and Gram-positive bacteria with MICs ranging from 2.5 mg/mL to 5 mg/mL [36]. Al-Janabi et al. [54] revealed that acetaminophen exhibits inhibitory activity against *S. aureus*, *Escherichia coli*, *Bacillus sp*, *Enterobacter spp.*, *Salmonella sp.,* and *Paracoccus sp*., with MICs ranging from 1.25 mg/mL to 2.5 mg/mL. Many authors have reported synergistic antibacterial effects of antipyretics with different antibiotics [36,55,56]. This research investigated for the first time the combined effects of ibuprofen, acetylsalicylic acid, and acetaminophen with EEPs. Interestingly, all of the drugs tested showed synergistic interactions with ethanolic propolis extracts. These results suggest that combining EEP with ibuprofen, acetylsalicylic acid, or acetaminophen could be useful as adjuvant therapy in staphylococcal infections. Although the growth inhibition of pathogens by antipyretics and other non-antibiotics mostly occurs at levels above therapeutic plasma concentrations, combining them with other antimicrobials significantly reduces the doses. Moreover, they may show potential in a topical application and the treatment of localized infection. According to Pina-Vaz et al. [57], ibuprofen reaches inhibitory doses in the patient’s urine and can help treat urinary tract infections caused by *Candida albicans*.

## 5. Conclusions

The most common and important application of the antimicrobial potential of propolis is the treatment of skin and wound infections. The findings of this study revealed important interactions of EEPs with several important disinfectants, namely, chlorhexidine, octenidine dihydrochloride, and 2-phenoxyethanol. Unfortunately, only in the case of 2-phenoxyethanol, the interaction was positive. The combination of EEPs with two other disinfectants resulted in an important decrease in antimicrobial activity. Both these results are important and should be taken into account in a clinical scenario when EEP is used as an agent for treating bacterial infections. On the other hand, all antipyretics tested considerably improved the activity of EEP, which is also important for the medical/veterinary application of EEPs. The outcomes of the study also support the hypothesis that the propolis’s antimicrobial effect is due to the combined (synergistic) action of several ingredients rather than the presence of one component of high antibacterial activity.

## Figures and Tables

**Table 1:** Antimicrobial activity of antipyretics against different strains of *S. aureus.*

Bacterial Strains	MIC and MBC Against Different Strains of Bacteria					
	Acetaminophen (µg/mL)		Ibuprofen (µg/mL)		Acetylsalicylic Acid (µg/mL)	
	MIC	MBC	MIC	MIC	MBC	MIC
*S. aureus* ATCC 25923	8000	>8000	500	4000	2000	4000
*S. aureus* ATCC 29213	>8000	>8000	500	4000	2000	4000
MSSA 1	>8000	>8000	500	2000	2000	4000
MSSA 2	8000	>8000	500	4000	1000	4000
MSSA 3	>8000	>8000	500	4000	2000	4000
MSSA 4	>8000	>8000	500	4000	1000	4000
MSSA 5	8000	>8000	500	4000	2000	4000
MRSA 1	8000	>8000	500	2000	2000	4000
MRSA 2	>8000	>8000	500	2000	2000	4000
MRSA 3	8000	>8000	500	1000	2000	4000

MIC–minimum inhibitory concentration; MBC–minimum bactericidal concentration; MSSA–methicillin-susceptible *Staphylococcus aureus*; MRSA—methicillin-resistant *Staphylococcus aureus*.

**Table 2:** Antimicrobial activity of antiseptics against different strains of *S. aureus.*

Bacterial Strains	MIC and MBC (µg/mL) against Different Strains of Bacteria					
	2-Phenoxyethanol (%(*v/v*))		Chlorhexidine (µg/mL)		Octenidine Dihydrochloride (µg/mL)	
	MIC	MBC	MIC	MBC	MIC	MBC
*S. aureus* ATCC 25923	0.312	1.250	0.100	0.100	0.400	0.400
*S. aureus* ATCC 29213	0.312	1.250	0.100	0.100	0.200	0.200
MSSA 1	0.312	0.312	0.100	0.100	0.400	0.400
MSSA 2	0.312	0.625	0.100	0.100	0.200	0.200
MSSA 3	0.312	0.625	0.100	0.100	0.200	0.200
MSSA 4	0.312	1.250	0.100	0.200	0.200	0.200
MSSA 5	0.312	1.250	0.100	0.200	0.200	0.200
MRSA 1	0.312	0.312	0.400	0.400	0.200	0.200
MRSA 2	0.312	0.625	0.100	0.100	0.200	0.200
MRSA 3	0.156	0.625	0.100	0.200	0.200	0.200

**Table 3:** The checkerboard analysis of drug interaction between EEPs and different antiseptic against *S. aureus* ATCC 25923.

Agent	MIC (µg/mL)/((%(*v/v*)) *		FIC	ΣFIC	Interpretation
	Alone	Combination			
EEP 1	128.000	16.000	0.125	0.375	Synergy
PE	0.312	0.080	0.250		
EEP 2	128.000	16.000	0.125	0.375	Synergy
PE	0.312	0.080	0.250		
EEP 3	256.000	16.000	0.063	0.187	Synergy
PE	0.312	0.040	0.125		
EEP 4	128.000	8.000	0.063	0.312	Synergy
PE	0.310	0.080	0.250		
EEP 1	128.000	128.000	1.000	≥9.000	Antagonism
C	0.100	≥0.800	≥8.000		
EEP 2	128.000	256.000	2.000	≥10.000	Antagonism
C	0.100	≥0.800	≥8.000		
EEP 3	256.000	256.000	1.000	≥9.000	Antagonism
C	0.100	≥0.800	≥8.000		
EEP 4	128.000	256.000	2.000	≥10.000	Antagonism
C	0.100	≥0.800	≥8.000		
EEP 1	128.000	128.000	1.000	≥5.000	Antagonism
OD	0.400	≥1.600	≥4.000		
EEP 2	128.000	128.000	1.000	≥5.000	Antagonism
OD	0.400	≥1.600	≥4.000		
EEP 3	256.000	512.000	2.000	≥6.000	Antagonism
OD	0.400	≥1.600	≥4.000		
EEP 4	128.000	64.000	0.500	≥4.500	Antagonism
OD	0.400	≥1.600	≥4.000		

* PE—%(*v/v*); EEP, C, OD—µg/mL. PE—2-phenoxyethanol, C—chlorhexidine, OD—octenidine dihydrochloride.

**Table 4:** The checkerboard analysis of drug interaction between EEPs and antipyretics (IB—ibuprofen, AS—acetylsalicylic acid, AM—acetaminophen) against *S. aureus* ATCC 25923.

Agent	MIC (µg/mL)		FIC	ΣFIC	Interpretation
	Alone	Combination			
EEP 1	128.000	16.000	0.125	0.375	Synergy
IB	500.000	125.000	0.250		
EEP 2	128.000	16.000	0.125	0.250	Synergy
IB	500.000	62.500	0.125		
EEP 3	256.000	32.000	0.125	0.187	Synergy
IB	500.000	31.300	0.062		
EEP 4	128.000	16.000	0.125	0.250	Synergy
IB	500.000	62.500	0.125		
EEP 1	128.000	16.000	0.125	0.250	Synergy
AS	2000.000	125.000	0.062		
EEP 2	128.000	32.000	0.250	0.281	Synergy
AS	2000.000	62.500	0.031		
EEP 3	256.000	16.000	0.062	0.078	Synergy
AS	2000.000	31.250	0.016		
EEP 4	128.000	8.000	0.062	0.125	Synergy
AS	2000.000	125.000	0.062		
EEP 1	128.000	16.000	0.125	0.141	Synergy
AM	8000.000	125.000	0.016		
EEP 2	128.000	8.000	0.062	0.094	Synergy
AM	8000.000	250.000	0.016		
EEP 3	256.000	64.000	0.250	0.312	Synergy
AM	8000.000	500.000	0.062		
EEP 4	128.000	8.000	0.062	0.094	Synergy
AM	8000.000	250.000	0.016		

**Table 5:** Antimicrobial activity of propolis polyphenols and polyphenols mixture against reference strains of *S. aureus.*

Phenolic Compounds	MIC and MBC (µg/mL) against Different Strains of Bacteria			
	*S. aureus* ATCC 25923		*S. aureus* ATCC 29213	
	MIC	MBC	MIC	MBC
Ferulic acid	>1024	>1024	>1024	>1024
Isoferulic acid	>1024	>1024	>1024	>1024
Caffeic acid	>1024	>1024	>1024	>1024
*p*-Coumaric acid	>1024	>1024	>1024	>1024
Apigenin	>1024	>1024	>1024	>1024
Chrysin	>1024	>1024	>1024	>1024
Quercetin	64	>1024	64	>1024
Kaempferol	32	>1024	32	>1024
Galangin	32	>1024	32	>1024
Pinocembrin	128	>1024	128	512
Pinostrombin	>1024	>1024	>1024	>1024
Pinobanksin	1024	>1024	1024	>1024
Sakuranetin	256	>1024	256	>1024
Polyphenols mixture	128	1024	128	256

**Table 6:** Antimicrobial activity of propolis polyphenols and polyphenols mixture against clinical isolates of *S. aureus* (MSSA and MRSA).

Phenolic Compounds	MIC/MBC	MIC and MBC (µg/mL) against Different Strains of Bacteria							
		MSSA 1	MSSA 2	MSSA 3	MSSA 4	MSSA 5	MRSA 1	MRSA 2	MRSA 3
Quercetin	MIC	128	64	64	64	64	32	64	64
	MBC	>1024	>1024	>1024	>1024	>1024	>1024	>1024	>1024
Kaempferol	MIC	>1024	>1024	>1024	32	32	32	>1024	>1024
	MBC	>1024	>1024	>1024	>1024	>1024	>1024	>1024	>1024
Galangin	MIC	64	32	32	32	32	32	32	32
	MBC	>1024	>1024	>1024	>1024	>1024	>1024	>1024	>1024
Pinocembrin	MIC	256	256	128	128	128	128	256	256
	MBC	>1024	>1024	>1024	>1024	>1024	>1024	>1024	>1024
Pinobanksin	MIC	>1024	>1024	1024	1024	>1024	1024	>1024	>1024
	MBC	>1024	>1024	>1024	>1024	>1024	>1024	>1024	>1024
Sakuranetin	MIC	256	256	256	256	256	256	256	512
	MBC	>1024	>1024	>1024	>1024	>1024	>1024	>1024	>1024
Polyphenols mixture	MIC	256	128	128	128	128	32	128	128
	MBC	1024	1024	>1024	512	512	256	512	512

## Data Availability

The data presented in this study are available on request from the corresponding author.

## References

[1] van de Sande-Bruinsma N., Fo Wong D.L. (2014). WHO European strategic action plan on antibiotic resistance: How to preserve antibiotics. J. Pediatr. Infect. Dis..

[2] Fischbach M.A., Walsh C.T. (2009). Antibiotics for emerging pathogens. Science.

[3] Lowy F.D. (1998). Staphylococcus aureus Infections. N. Engl. J. Med..

[4] Plata K., Rosato A.E., Węgrzyn G. (2009). Staphylococcus aureus as an infectious agent: Overview of biochemistry and molecular genetics of its pathogenicity. Acta Biochim. Pol..

[5] Mueller E., Haim M., Petnehazy T., Acham-Roschitz B., Trop M. (2010). An innovative local treatment for staphylococcal scalded skin syndrome.Eur. J. Clin. Microbiol. Infect. Dis..

[6] Kulhankova K., King J., Salgado-Pabon W. (2014). Staphylococcal toxic shock syndrome: Superantigen-mediated enhancement of endotoxin shock and adaptive immune suppression. Immunol. Res..

[7] Fisher E.L., Otto M., Cheung G.Y.C. (2018). Basis of Virulence in Enterotoxin-Mediated Staphylococcal Food Poisoning. Front. Microbiol..

[8] Wise R. (2011). BSAC Working Party on The Urgent Need: Regenerating Antibacterial Drug Discovery and Development. The urgent need for new antibacterial agents. J. Antimicrob. Chemother..

[9] Wink M. (2008). Evolutionary advantage and molecular modes of action of multi-component mix-tures used in phytomedicine. Curr. Drug Metab..

[10] Wink M. (2015). Modes of action of herbal medicines and plant secondary metabolites. Medicines.

[11] Szweda P. (2017). Antimicrobial Activity of Honey. Agricultural and Biological Sciences: Honey Analysis.

[12] Szweda P., Kot B. (2017). Bee Products and Essential Oils as Alternative Agents for Treatment of Infections Caused by *S. aureus*. Frontiers in Staphylococcus aureus.

[13] Huang S., Zhang C.-P., Wang K., Li G., Hu F.-L. (2014). Recent Advances in the Chemical Composition of Propolis. Molecules.

[14] Kujumgiev A., Tsvetkova I., Serkedjieva Y., Bankova V., Christov R., Popov S. (1999). Antibacterial, antifungal and antiviral activity of propolis of different geographic origin. J. Ethnopharmacol..

[15] Popova M., Bankova V., Butovska D., Petkov V., Nikolova-Damyanova B., Sabatini A.G., Marcazzan G.L., Bogdanov S. (2004). Validated Methods for the Quantification of Biologically Active Constituents of Poplar-type Propolis. Phytochem. Anal..

[16] Wojtyczka R.D., Dziedzic A., Idzik D., Kepa M., Kubina R., Kabała-Dzik A., Smoleń-Dzirba J., Stojko J., Sajewicz M., Wasik T.J. (2013). Susceptibility of Staphylococcus aureus clinical isolates to propolis extract alone or in combination with antimicrobial drugs. Molecules.

[17] Grecka K., Kuś P.M., Okińczyc P., Worobo R.W., Walkusz J., Szweda P. (2019). The anti-staphylococcal potential of ethanolic Polish propolis extracts. Molecules.

[18] Przybyłek I., Karpiński T.M. (2019). Antibacterial Properties of Propolis. Molecules.

[19] Grecka K., Xiong Z.R., Chen H., Pełka K., Worobo R.W., Szweda P. (2020). Effect of Ethanol Extracts of Propolis (EEPs) against Staphylococcal Biofilm—Microscopic Studies. Pathogens.

[20] Haghdoost N.S., Salehi T.Z., Khosravi A., Sharifzadeh A. (2016). Antifungal activity and influence of propolis against germ tube formation as a critical virulence attribute by clinical isolates of Candida albicans. J. Mycol. Méd..

[21] Gucwa K., Kusznierewicz B., Milewski S., van Dijck P., Szweda P. (2018). Antifungal Activity and Synergism with Azoles of Polish Propolis. Pathogens.

[22] Inui S., Hatano A., Yoshino M., Hosoya T., Shimamura Y., Masuda S., Ahn M.-R., Tazawa S., Araki Y., Kumazawa S. (2014). Identification of the phenolic compounds contributing to antibacterial activity in ethanol extracts of Brazilian red propolis. Nat. Prod. Res..

[23] Cushnie T.P.T., Lamb A.J. (2005). Antimicrobial activity of flavonoids. Int. J. Antimicrob. Agents.

[24] Mirzoeva O.K., Grishanin R.N., Calder P.C. (1997). Antimicrobial action of propolis and some of its components: The effects on growth, membrane potential and motility of bacteria. Microbiol. Res..

[25] Al-Waili N., Al-Ghamdi A., Ansari M.J., Al-Attal Y., Salom K. (2012). Synergistic effects of honey and propolis toward drug multi-resistant Staphylococcus Aureus, Escherichia coli and Candida Albicans isolates in single and polymicrobial cultures. Int. J. Med. Sci..

[26] Scazzocchio F., D’Auria F.D., Alessandrini D., Pantanella F. (2006). Multifactorial aspects of antimicrobial activity of propolis. Microbiol. Res..

[27] Speciale A., Costanzo R., Puglisi S., Musumeci R., Catania M.R., Caccamo F., Iauk L. (2006). Antibacterial activity of propolis and its active principles alone and in combination with macrolides, beta-lactams and fluoroquinolones against microorganisms responsible for respiratory infections. J. Chemother..

[28] de Oliveira Orsi R., Maurício Sforcin J., Regina Cunha Funari S., Fernandes Junior A., Bankova V. (2006). Synergistic effect of propolis and antibiotics on the Salmonella Typhi. Braz. J. Microbiol..

[29] Fernandes Júnior A., Cristina Balestrin E., Elaine Cristina Betoni J., de Oliveira Orsi R., de Lourdes Ribeiro de Souza da Cunha M., Cezar Montelli A. (2005). Propolis: Anti-Staphylococcus aureus activity and synergism with antimicrobial drugs. Mem. Inst. Oswaldo Cruz.

[30] Stepanović S., Antić N., Dakić I., Svabić-Vlahović M. (2003). In vitro antimicrobial activity of propolis and synergism between propolis and antimicrobial drugs. Microbiol. Res..

[31] Orsi R.O., Fernandes A., Bankova V., Sforcin J.M. (2012). The effects of Brazilian and Bulgarian propolis in vitro against Salmonella Typhi and their synergism with antibiotics acting on the ribosome. Nat. Prod. Res..

[32] Nostro A., Cellini L., di Bartolomeo S., Cannatelli M.A., di Campli E., Procopio F., Grande R., Marzio L., Alonzo V. (2006). Effects of combining extracts (from propolis or Zingiber officinale) with clarithromycin on Helicobacter pylori. Phytother. Res..

[33] Ong T.H., Chitra E., Ramamurthy S., Ling C.C.S., Ambu S.P., Davamani F. (2019). Cationic chitosan-propolis nanoparticles alter the zeta potential of S. Epidermidis, inhibit biofilm formation by modulating gene expression and exhibit synergism with antibiotics. PLoS ONE.

[34] Vermeulen H., Westerbos S.J., Ubbink D.T. (2010). Benefit and harm of iodine in wound care: A systematic review. J. Hosp. Infect..

[35] Zimmermann P., Curtis N. (2017). Antimicrobial Effects of Antipyretics. Antimicrob. Agents Chemother..

[36] Chan E.W.L., Yee Z.Y., Raja I., Yap J.K.Y. (2017). Synergistic effect of non-steroidal anti-inflammatory drugs (NSAIDs) on antibacterial activity of cefuroxime and chloramphenicol against methicillin-resistant Staphylococcus aureus. J. Glob. Antimicrob. Resist..

[37] Foerster S., Desilvestro V., Hathaway L.J., Althaus C.L., Unemo M. (2017). A new rapid resazurin-based microdilution assay for antimicrobial susceptibility testing of Neisseria gonorrhoeae. J. Antimicrob. Chemother..

[38] Odds F.C. (2003). Synergy, antagonism, and what the chequerboard puts between them. J. Antimicrob. Chemother..

[39] di Veroli G.Y., Fornari C., Wang D., Mollard S., Bramhall J.L., Richards F.M., Jodrell D.I. (2016). Combenefit: An interactive platform for the analysis and visualization of drug combinations. Bioinformatics.

[40] Takaisi-Kikuni N., Schilcher H. (1994). Electron Microscopic and Microcalorimetric Investigations of the Possible Mechanism of the Antibacterial Action of a Defined Propolis Provenance. Planta Med..

[41] Bryan J., Redden P., Traba C. (2016). The mechanism of action of Russian propolis ethanol extracts against two antibiotic-resistant biofilm-forming bacteria. Lett. Appl. Microbiol..

[42] Castaldo S., Capasso F. (2002). Propolis, an old remedy used in modern medicine. Fitoterapia.

[43] Wang T., Li Q., Bi K. (2018). Bioactive flavonoids in medicinal plants: Structure, activity and biological fate. Asian J. Pharm. Sci..

[44] Ramadhan F., Mukarramah L., Oktavia F.A.R.H., Yulian R., Annisyah N.H., Asyiah I.N. (2018). Flavonoids from endophytic bacteria of cosmos caudatus Kunth. Leaf as anticancer and antimicrobial. Asian J. Pharm. Clin. Res..

[45] Havsteen B. (1983). Flavonoids, a class of natural products of high pharmacological potency. Biochem. Pharmacol..

[46] Eagle H., Musselman A.D. (1948). The rate of bactericidal action of penicillin in vitro as a function of its concentration, and its paradoxically reduced activity at high concentrations against certain organisms. J. Exp. Med..

[47] Langsrud S., Steinhauer K., Lüthje S., Weber K., Goroncy-Bermes P., Holck A.L. (2016). Ethylhexylglycerin Impairs Membrane Integrity and Enhances the Lethal Effect of Phenoxyethanol. PLoS ONE.

[48] Lim K.-S., Kam P.C.A. (2008). Chlorhexidine-Pharmacology and Clinical Applications. Anaesth. Intensive Care.

[49] Hübner N.-O., Siebert J., Kramer A. (2010). Octenidine Dihydrochloride, a Modern Antiseptic for Skin, Mucous Membranes and Wounds. Ski. Pharmacol. Physiol..

[50] Obad J., Šušković J., Kos B. (2015). Antimicrobial activity of ibuprofen: New perspectives on an “Old” non-antibiotic drug. Eur. J. Pharm. Sci..

[51] Lee C.-H., Su L.-H., Liu J.-W., Chang C.-C., Chen R.-F., Yang K.-D. (2014). Aspirin enhances opsonophagocytosis and is associated to a lower risk for Klebsiella pneumoniaeinvasive syndrome. BMC Infect. Dis..

[52] Kalle A.M., Rizvi A. (2011). Inhibition of Bacterial Multidrug Resistance by Celecoxib, a Cyclooxygenase-2 Inhibitor. Antimicrob. Agents Chemother..

[53] Yin Z., Wang Y., Whittell L.R., Jergic S., Liu M., Harry E., Dixon N.E., Kelso M.J., Beck J.L., Oakley A.J. (2014). DNA Replication Is the Target for the Antibacterial Effects of Nonsteroidal Anti-Inflammatory Drugs. Chem. Biol..

[54] Hussein A., AL-Janabi S. (2010). In Vitro antibacterial activity of ibuprofen and acetaminophen. J. Glob. Infect. Dis..

[55] Gil D., Daffinee K., Friedman R., Bhushan B., Muratoglu O.K., LaPlante K., Oral E. (2020). Synergistic antibacterial effects of analgesics and antibiotics against Staphylococcus aureus. Diagn. Microbiol. Infect. Dis..

[56] Altaf M., Ijaz M., Ghaffar A., Rehman A., Avais M. (2019). Antibiotic susceptibility profile and synergistic effect of non-steroidal anti-inflammatory drugs on antibacterial activity of resistant antibiotics (Oxytetracycline and Gentamicin) against methicillin resistant Staphylococcus aureus (MRSA). Microb. Pathog..

[57] Pina-Vaz C., Sansonetty F., Rodrigues A.G., Martinez-DE-Oliveira J., Fonseca A.F., Mårdh P.-A. (2000). Antifungal activity of ibuprofen alone and in combination with fluconazole against Candida species. J. Med. Microbiol..

